# Biliary stent migration complicated by sigmoid colon perforation with iliopsoas and acetabular involvement

**DOI:** 10.1007/s11845-026-04345-8

**Published:** 2026-04-01

**Authors:** İrem Ceren Koç, Mehmet Selim Nural, Gülten Taşkın

**Affiliations:** Faculty of Medicine, Department of Radiology, Ondokuz Mayıs University, Samsun, Turkey

**Keywords:** Biliary stent, Stent migration, Iliopsoas abscess, Colon perforation, Acetabular erosion

## Abstract

Biliary stenting is a common treatment for obstructive jaundice in patients with benign or malignant biliary pathologies, particularly when surgical intervention is high-risk due to comorbidities. Although generally safe, stent-related complications can occur. They are typically classified as early or late. Stent migration is one such complication and is often asymptomatic. However, in rare instances it can lead to gastrointestinal perforation. We present a highly unusual case of a migrated biliary stent that perforated the sigmoid colon, traversed the lateral pelvic wall, and focally eroded the anterior acetabulum, resulting in acetabular erosion and an iliopsoas abscess. To our knowledge, this is the first reported case with such a trajectory and complication profile. Clinicians and radiologists should consider the possibility of stent-related gastrointestinal and even musculoskeletal involvement, particularly in patients presenting with unexplained pain and a history of biliary stenting.

## Introduction

Biliary stenting is a widely used therapeutic procedure in cases of obstructive jaundice for both benign and malignant pathologic processes in the hepatobiliary tree and pancreas [1]. The procedure of endoscopic placement of biliary stents was first described by Soehendra and Reynder-Frederix in 1980, and it rarely causes complications [2, 3]. Complications related to biliary stents are divided into two categories: early complications and late complications. Local hemorrhage, transient hemobilia, and malposition are early complications. Bleeding, stent occlusion, and fistulation are late complications. Stent migration and perforation are considered in both the early and late complication groups [4].

Biliary stent migration is found to happen in only 6% of cases [5]. Dilated common bile ducts and longer biliary stents are risk factors for stent migration [6]. Most of the time, migratory biliary stents navigate the gastrointestinal tract and exit the body without any complications [4, 5]. Perforation due to migrated biliary stents is very rare. The most common site of perforation is the duodenum. The colon is an even rarer site of perforation [6, 7].

Biliary stent migration can cause various complications, but musculoskeletal involvement such as iliopsoas abscess is extremely rare. We report the first known case of a migrated biliary stent that perforated the sigmoid colon, traversed the lateral pelvic wall, and focally eroded the anterior acetabulum, causing bony erosion and an iliopsoas abscess. Although rare, radiologists should be aware of these potential musculoskeletal complications.

## Case report

A 64-year-old female patient was admitted to our emergency unit with complaints of abdominal pain and left hip and leg pain. Her known diseases were congestive heart failure, hypertension, chronic obstructive lung disease, and intracranial aneurysm. She had a history of cerebrovascular event and usage of anticoagulant drugs since then. She also had a known history of choledocholithiasis. When her previous admissions to our hospital were checked, she was admitted for right upper quadrant pain in April 2023. Her lab results then showed increased CRP (111), GGT (689 IU/L), AST (183 IU/L), ALT (239 IU/L), direct bilirubin (5.0 mg/dL), and total bilirubin (5.27 mg/dL). Her hepatobiliary ultrasound exam at the time revealed a gallbladder with increased diameter, stones in the lumen, and a widened common biliary duct (13.5 mm). Because she also had stones in intrahepatic biliary ducts, endoscopic retrograde cholangiopancreatography (ERCP) with a 12-centimeter 10 F plastic stent was done. After stent placement, her symptoms lessened. Her CRP (31.5), GGT (155 IU/L), AST (13.4 IU/L), ALT (23.1 IU/L), direct bilirubin (0.44 mg/dL), and total bilirubin (0.74 mg/dL) regressed, and she was discharged. She was admitted in August 2024 due to an increase in left leg and hip pain. As she had a chronic complaint of back, hip, and leg pain, a special focus was not given to the left leg and hip at the time. She had symptomatic treatment in the algology department and was discharged. In September 2024, her right upper quadrant abdominal pain complaint recurred and showed a gradual increase. She visited another hospital, and the workup of hepatobiliary ultrasound and contrast-enhanced upper abdomen CT in that center revealed that her biliary stent was not located in the common biliary duct. Her lab results showed increased CRP (92), AST (140.5 IU/L), ALT (76.4 IU/L), direct bilirubin (3.72 mg/dL), and total bilirubin (5.38 mg/dL). Thus, she had a repeat ERCP, and her symptom of abdominal pain decreased. Her CRP (5), AST (13.4 IU/L), ALT (16.4 IU/L), direct bilirubin (0.61 mg/dL), and total bilirubin (1.32 mg/dL) regressed. In November 2024—her current admittance was for a gradual increase in abdominal, left leg, and left hip pain. Her physical exam showed pretibial edema, decreased movement of the left hip, and general tenderness in the abdomen. She didn’t have a fever; her vitals were within normal range. Her lab results revealed a normal WBC (8.52 × 10^9^ L), AST (16 IU/L), ALT (9 IU/L), and GGT (106 IU/L) and an increased direct bilirubin (1.65 mg/dL), total bilirubin (2.32 mg/dL), and CRP (108). A hip radiography and a contrast-enhanced abdominal CT were done.

**Fig. 1:**
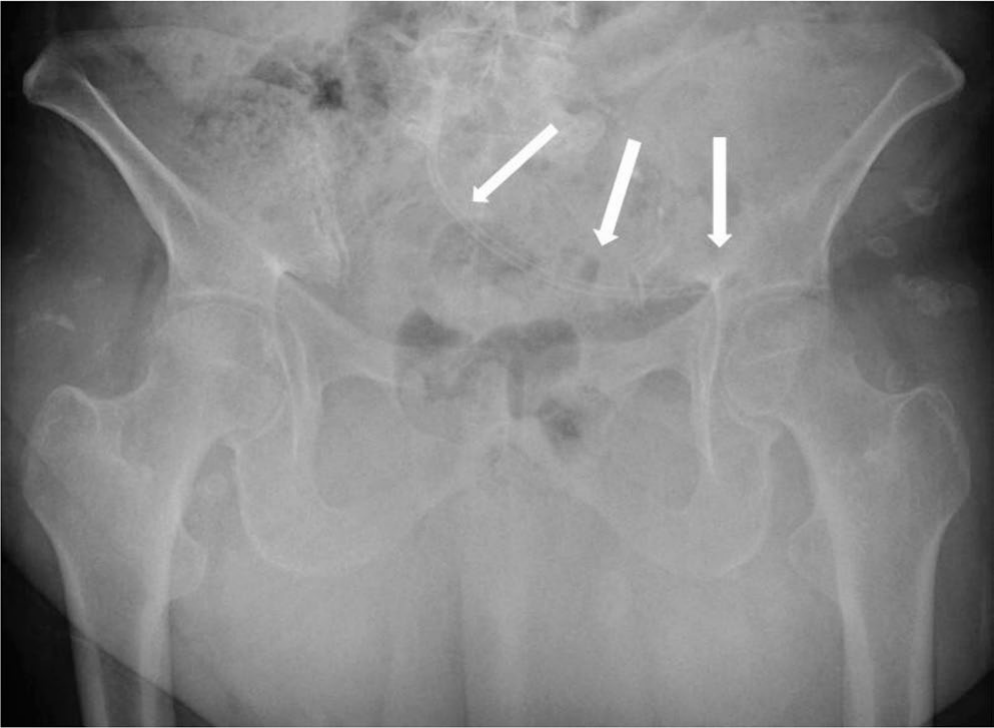
An anteroposterior hip radiograph. A curvilinear biliary stent is seen extending from the mid-pelvis into the left lower quadrant, passing over the iliopectineal and ilioischial lines, with the distal lateral end of the stent terminating at the left acetabular roof (arrows)

**Fig. 2:**
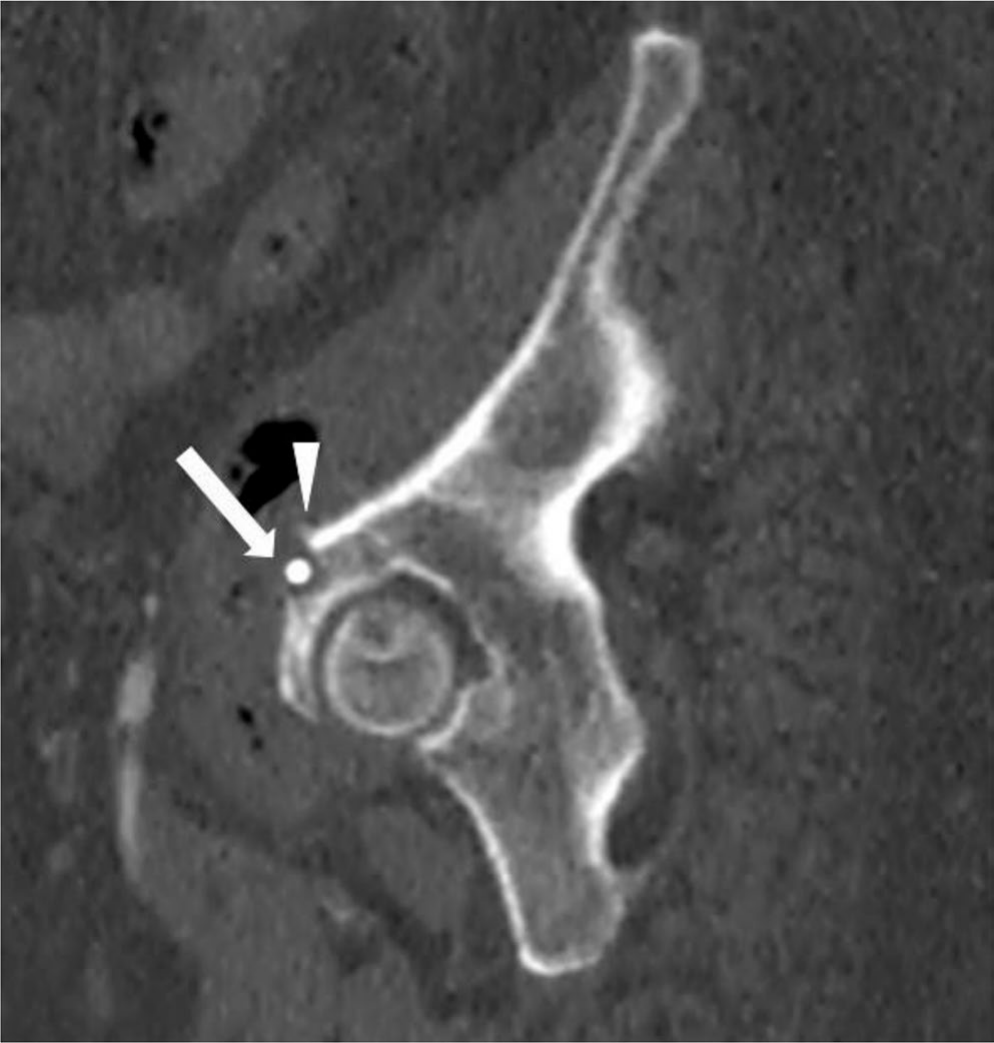
Sagittal contrast-enhanced CT of the pelvis in bone window. A migratory stent is seen causing focal erosion in the anteromedial aspect of the acetabular roof, with sharp margins and a smooth contour, extending into the central medullary portion of the iliac bone (arrow). A single-layered, linear periosteal reaction is observed in a small area immediately adjacent (arrowhead)

**Fig. 3:**
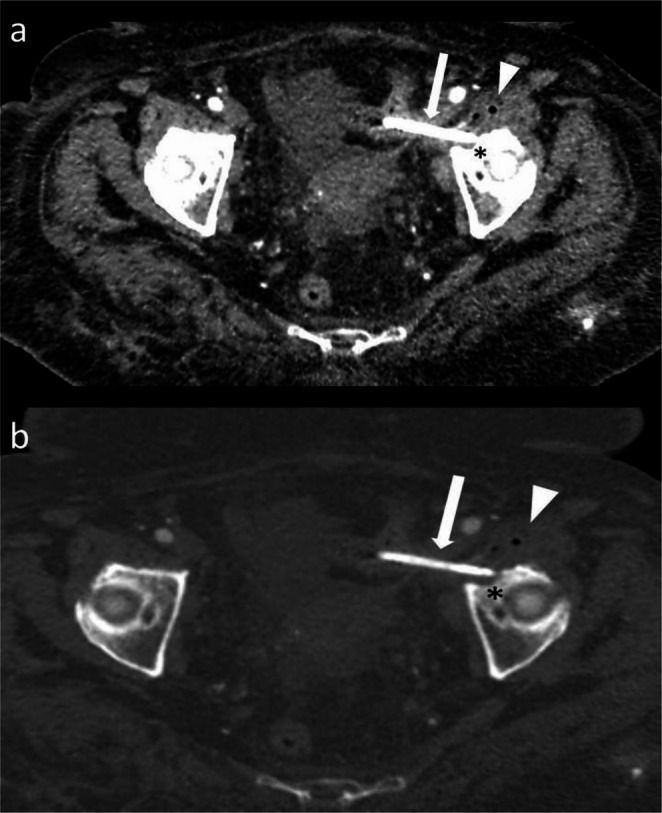
Axial contrast-enhanced CT of the pelvis in soft tissue window (**a**) and bone window (**b**). A biliary stent perforating the sigmoid colon wall (arrow), a left iliopsoas muscle abscess (arrowhead), and focal erosion on the acetabulum (*) are seen. Peripherally, sclerotic changes extending toward the anterior acetabular roof are observed in the ilium, along with cortical irregularities and small erosions with sclerotic borders

Radiographic evaluation demonstrated a curvilinear biliary stent extending from the mid-pelvis into the left lower quadrant, passing over the iliopectineal and ilio-ischial lines. Even on a single-projection radiograph, the distal lateral end of the stent could be clearly identified, terminating at the left acetabular roof. The presence of limited focal osteolytic changes in the regions adjacent to the acetabular component supported the interpretation that the stent was in direct relation with the bone. No communication with the hip joint was evident, and the radiographs showed no periosteal reaction, broad loss of cortical definition, or sclerosis (Fig. 1). Contrast-enhanced CT of the abdomen and pelvis showed a correctly placed stent in the common biliary duct and a migratory stent in the distal sigmoid colon. The migratory stent was positioned partially within the lumen of the sigmoid colon. The remaining segment perforated the colonic wall and passed through the fat plane, left iliopsoas fascia, and iliac muscle, crossing the arcuate line of the ilium. This resulted in a focal erosion in the anteromedial aspect of the acetabular roof, with sharp margins and a smooth contour, extending into the central medullary portion of the iliac bone. The sharply demarcated margins of the erosion and its direct apposition to the stent were consistent with pressure-induced erosion. A single-layered, linear periosteal reaction was observed in a small area immediately adjacent (Fig. 2). More peripherally, sclerotic changes extending toward the anterior acetabular roof were observed in the ilium, along with cortical irregularities and small erosions with sclerotic borders. The observed findings lacked typical features of acute or chronic osteomyelitis and were instead interpreted as focal erosions with associated edematous bone marrow changes, secondary to chronic iliopsoas abscess formation or stent-related local irritation (Fig. 3a).

**Fig. 4:**
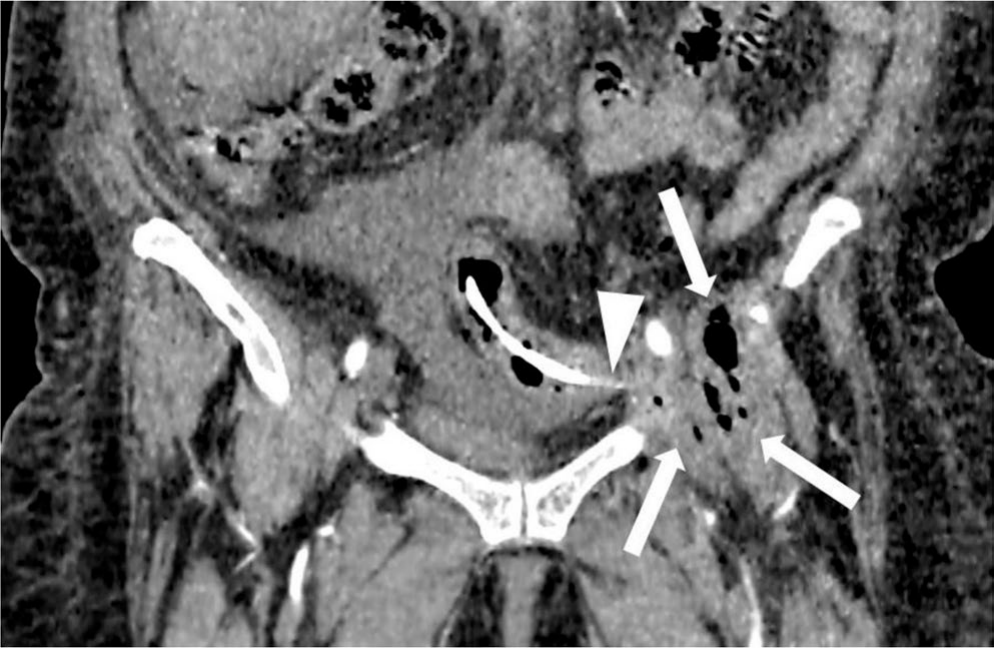
Coronal contrast-enhanced CT of the pelvis in soft tissue window. A biliary stent perforating the sigmoid colon wall (arrowhead) is seen. At the level of the migratory stent, a 38 × 25 mm abscess with a thick wall within the iliopsoas muscle, containing multiple foci of air and demonstrating peripheral contrast enhancement, is observed (arrows). The abscess is extending inferiorly along the iliopsoas bursa

At the level of the migratory stent, a 38 × 25 mm multiseptated abscess with a thick wall was identified within the iliopsoas muscle, containing multiple foci of air and demonstrating peripheral contrast enhancement. The abscess was closely opposed to the iliac cortical bone, which remained largely intact without evidence of cortical destruction and extended inferiorly along the iliopsoas bursa (Figs. 3b and 4). The sigmoid colon’s perforated wall and the lateral wall of the pelvis were adhered together posterior to the left external iliac artery and vein. The migratory stent caused a thin fistula between these two structures, and the perforation was self-limited. Perihepatic, perisplenic, paracolic, and pelvic ascites were present due to her congestive heart failure, and were the main cause of her general abdominal pain. A multidisciplinary team including infectious disease, orthopedics, and general surgery determined that, given the patient’s lack of acute peritonitis, self-limited perforation, and her comorbid conditions, surgical intervention was not warranted. Interventional radiology was consulted for the abscess, but due to localization and comorbidities, abscess drainage was not done. As for left hip and left leg pain, an observational approach was decided upon, the first being the treatment of abdominal pathologies and seeing if the pain resolved after inflammation control. Conservative management was initiated, including empiric antibiotics (amoxicillin clavulanate 3 × 1 g and ciprofloxacin 2 × 500 mg per oral for 6 weeks) and pain control (naproxen sodium per oral when needed). Stent removal via rectosigmoidoscopy was decided upon as treatment. The procedure done for treatment also confirmed our diagnosis.

## Discussion

Perforation due to migratory biliary stent is not expected. When perforation happens, the small bowel is the common site, with the duodenum being the primarily affected part. In a study done by Zorbas et al., there were 81 cases reported of small bowel perforation due to migratory biliary stent between 2000 and 2020 [7]. Colonic perforation is an even rarer complication. There were only 37 cases reported between the years 1993 and 2022, according to a study done by Wilson et al. Colonic perforation due to migratory biliary stents usually presents with abdominal pain. If not recognized early on, perforation can result in mortality [6].

Our patient’s CT findings revealed a migrated stent that perforated the sigmoid colon, crossed the iliac arcuate line, and focally eroded the anteromedial aspect of the acetabulum. The left iliopsoas muscle abscess at the level of the stent accompanied the sigmoid colon perforation. The combination of acetabular surface erosion, adjacent sclerotic changes, a subtle linear periosteal reaction, preserved cortical integrity, and the presence of a dense multiseptated abscess were strongly indicative of a chronic pathologic process. Overall, the radiologic findings were consistent with the hypothesis that bone erosion, initially triggered by pressure effects from the foreign body, was further facilitated by inflammatory changes associated with the concomitant soft tissue infection, thereby promoting bone resorption. Her leg and hip pain were attributed mainly to the inflammation caused by iliopsoas alongside these processes. Importantly, her presentation was subtle, with minimal abdominal signs despite perforation, highlighting the need for a high index of suspicion.

To our knowledge, only one previous case has been reported of a migrated biliary stent causing sigmoid colon perforation and iliopsoas abscess. However, this is the first documented instance of a biliary stent going through the fat plane, left iliopsoas fascia, and iliac muscle and focally eroding the anterior acetabulum, making this an extremely rare presentation [8].

In conclusion, while biliary stent migration is uncommon, its complications can be severe. Clinicians and radiologists should consider the possibility of stent-related gastrointestinal and even musculoskeletal involvement, particularly in patients presenting with unexplained pain and a history of biliary stenting. Early recognition, and coordinated multidisciplinary management are essential to reduce morbidity.

## Data Availability

The data supporting the findings of this case report are not publicly available due to concerns regarding patient privacy and confidentiality. Further details are available from the corresponding author upon reasonable request.
